# Quadrilateral Micro-Hole Array Machining on Invar Thin Film: Wet Etching and Electrochemical Fusion Machining

**DOI:** 10.3390/ma11010160

**Published:** 2018-01-19

**Authors:** Woong-Kirl Choi, Seong-Hyun Kim, Seung-Geon Choi, Eun-Sang Lee

**Affiliations:** Department of Mechanical Engineering, Inha University, Incheon 402-751, Korea; xiongjie_123@hotmail.com (W.-K.C.); bombbb@hanmail.net (S.-H.K.); tlohhero@naver.com (S.-G.C.)

**Keywords:** wet etching, electrochemical machining, fusion machining, Invar film, micro-hole array

## Abstract

Ultra-precision products which contain a micro-hole array have recently shown remarkable demand growth in many fields, especially in the semiconductor and display industries. Photoresist etching and electrochemical machining are widely known as precision methods for machining micro-holes with no residual stress and lower surface roughness on the fabricated products. The Invar shadow masks used for organic light-emitting diodes (OLEDs) contain numerous micro-holes and are currently machined by a photoresist etching method. However, this method has several problems, such as uncontrollable hole machining accuracy, non-etched areas, and overcutting. To solve these problems, a machining method that combines photoresist etching and electrochemical machining can be applied. In this study, negative photoresist with a quadrilateral hole array pattern was dry coated onto 30-µm-thick Invar thin film, and then exposure and development were carried out. After that, photoresist single-side wet etching and a fusion method of wet etching-electrochemical machining were used to machine micro-holes on the Invar. The hole machining geometry, surface quality, and overcutting characteristics of the methods were studied. Wet etching and electrochemical fusion machining can improve the accuracy and surface quality. The overcutting phenomenon can also be controlled by the fusion machining. Experimental results show that the proposed method is promising for the fabrication of Invar film shadow masks.

## 1. Introduction

Invar is a 36% nickel iron alloy which has the lowest thermal expansion among all metals and alloys in the range from room temperature up to approximately 230 °C. This value is 10 times lower than that of stainless steel and 100 times lower than that of iron, which makes Invar appropriate for application in various precision fields. For instance, Invar is used where high dimensional stability is required, such as clocks, seismic creep gauges, organic light-emitting diode (OLED) display shadow masks, values in engines, antimagnetic watches, and controllers for the quality, color, and definition of an image on mobile devices. Additionally, because of small dimensional changes due to temperature, Invar is ideally suited to aerospace tooling [1,2].

Mechanical processes, etching, and electroforming are currently the most-used machining methods for Invar micro-feature devices, although other methodologies have been suggested [3,4]. Advanced hole machining methods are being studied by many researchers for maintaining Invar thin film arresting feature, such as electro-discharge machining (EDM), laser beam machining (LBM), electron beam machining (EBM), and electrochemical machining (ECM). However, the formation of heat-affected zones and micro-cracks on the workpieces could occur, because EDM, LBM, and EBM are all thermal processes [5,6,7].

The shadow mask is one of the most important factors for patterning OLED pixels, and it is becoming more important as the panel resolution and generation rises. It is a particular metal film that is used in high-resolution mobile and tablet displays’ deposition industries. It is used to deposit organic compounds to realize the RGB (Red, Green, Blue) color of OLEDs. Shadow masks are mainly made from Invar alloy because of its low thermal expansion coefficient, strength at extremely low temperature, and impact toughness. The machined holes’ accuracy influences the quality of the display resolution, and therefore the machined hole accuracy and uniformity are vital factors in shadow mask fabrication.

For Invar thin film machining with thickness less than 50 µm, laser machining, electroforming, and wet etching methods are mostly used in the field. However, laser machining has problems with burrs, which are inappropriate for precision machining, and electroforming has short component service life and high production cost problems. Wet etching is the most commonly used method for machining Invar thin film holes because of its high material removal rate and high efficiency. However, because of the isotropic machining characteristics, there are undercutting characteristics in this method. Furthermore, the adhesion rate between the film and DFR (Dry Film Resist) coating could decrease as the machining time increases. Therefore, controlling the machining geometry in wet etching is a very challenging task.

ECM is a method of removing workpiece metal by a controlled anodic dissolution; the workpiece acts as the anode, and the tool works as a cathode in the electrolyte. The electrolyte should be supplied to the machining zone at a set temperature during the machining process. The workpiece is eroded in accordance with Faraday’s law, and the tool forms the desired shape in the workpiece. ECM does not induce any mechanical or thermal stress, and it does not alter the surface’s chemical composition; additionally, mirror surface finishes can be achieved [8,9,10,11,12]. It is receiving more and more attention for microfabrication, and is one of the appropriate methods for Invar film hole machining.

In this paper, photoresist single-side wet etching and a fusion method of wet etching and ECM were used to machine micro-holes on 30-µm-thick Invar thin film, which was dry coated using a negative photoresist with a quadrilateral hole array. Because of the isotropic characteristics of wet etching, the hole geometry is difficult to control. Thus, the fusion method was investigated to obtain an accurate geometry hole array. Two types of dipping wet etching were carried out before ECM: etching on the photoresist-coated face and etching on the uncoated face. After wet etching, ECM was used to machine a micro-hole array. The experimental results show that thinning the Invar film using wet etching on the uncoated face before ECM is appropriate for micro-hole array machining. ECM also improves the film surface quality, which is an important factor for shadow masks. 

## 2. Experimental Details

### 2.1. Invar Thin Film DFR Coating

**Figure 1:**
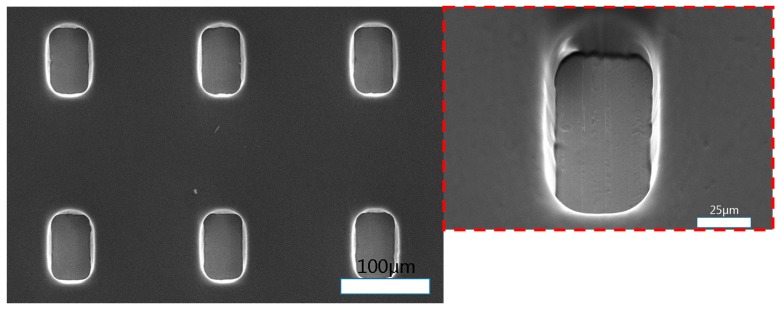
SEM image of DFR (Dry Film Resist) -coated Invar thin film.

**Figure 2:**
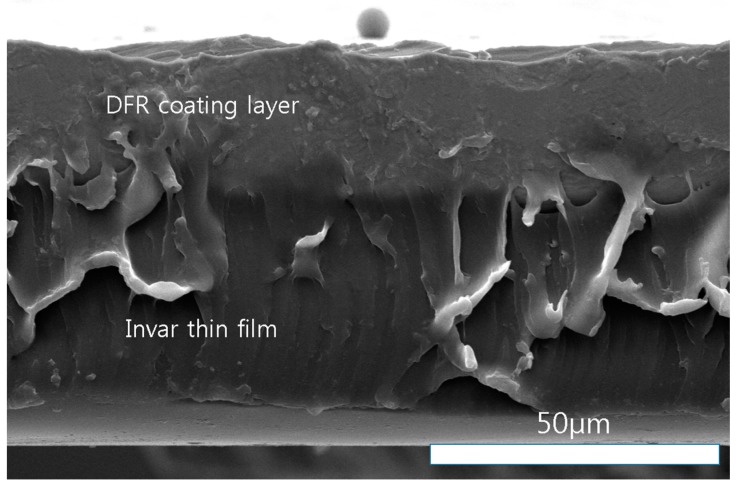
Sectional SEM image of DFR coated Invar thin film.

Figure 1 shows an SEM (Scanning Electron Microscope) image of DFR-coated Invar thin film. The photoresist is the negative type, and the thickness of the DFR film (Kolon Corp., Gwacheon-si, Korea) is 30 µm, as shown in Figure 2. The Invar film surface was rinsed using DI water for 60 s and dried using nitrogen gun, and then normal lamination was carried out. The DFR roll temperature was 100 °C, the roll speed was 2 m/min, and the roll pressure was 0.4 MPa. After the lamination, the sample was held at 20 °C for 20 min. 

The mask used in the exposure was a film mask with a 60 × 90 µm quadrilateral array. The developer was an aqueous solution of 2 wt % Na_2_CO_3_ solution, and the temperature was 30 °C. After development, the Invar film was sprayed with water and baked (100 °C, 60 s) to improve the adhesion rate between the Invar and DFR. The adhesion is very important for improving the machining geometry and decreasing overcutting. 

### 2.2. Wet Etching 

**Figure 3:**
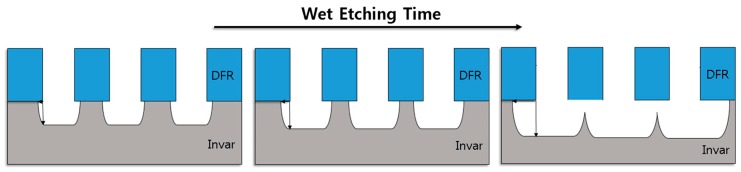
Invar film wet etching isotropic characteristics.

Wet etching in the semiconductor industry commonly involves etchants with wafers immersed in etchant solution. This method is cheap and simple, but is difficult to control. Therefore, it is not popular in nanofabrication for pattern transfer purposes. Because of the isotropic etching results, it is best to use this method for large features when the sidewall slope does not matter and to undercut the mask. Figure 3 shows the Invar film isotropic wet etch characteristics according to the etching time.

In spite of the isotropic etch characteristics, wet etching is used for Invar film hole machining because of its high machining efficiency. However, the yield for Invar OLED shadow mask fabrication is very low due to the issues of control and isotropic characteristics. Additionally, the hole size is difficult to decrease, because the Invar and photoresist could be separated before generating the hole due to undercutting. In Invar shadow mask wet etching fabrication, decrease of Invar film thickness could increase the machining accuracy and geometry. However, the manufacturing of an Invar thin film of under 30 µm thickness is very difficult in the field. Further, both side spray-type wet etching is also used in the field by laminating a double-sided DFR pattern using alignment technology. This technology needs more time and high-level alignment and exposure technology compared to one-face DFR patterning. Therefore, the study on efficient and convenient Invar film hole machining methods on a 30 µm-thick substrate is ongoing, and one of the most challenging research topics is increasing the yield of Invar shadow mask fabrication using simple machining methods. 

### 2.3. Electrochemical Machining 

For machining heat-resistant and high-strength materials into complex shapes, compared to conventional machining methods, ECM has been found to be economical and effective. In recent years, ECM has been applied to various industries (e.g., the automobile and turbo-machinery industries) for its advantages, such as no tool wear and the ability to machine difficult-to-cut metals with complex geometries and relatively high accuracy. In the machining of metal components which need high precision, accuracy is the most important factor, and many studies have been carried out to improve it. Such studies have investigated the effectiveness of forming smaller gaps between the cathode and anode, the application of dual-pole electrodes, orbital electrode movement, ultrasonically-assisted ECM, a new method for monitoring the inter-electrode gap, the influence of electrochemical micro-drilling by short-pulse voltages, and modelling of the ECM process by the boundary element method. The optimization of machining parameters has also been studied. Other techniques in ECM include the use of a low-concentration electrolyte and acidified sodium chloride electrolyte, a two-dimensional two-phase flow field for tool design, a CAE (Computer Aided Engineering)—ECM system, ECM of a spiral internal turbulator, and laser-assisted jet ECM [13]. 

**Figure 4:**
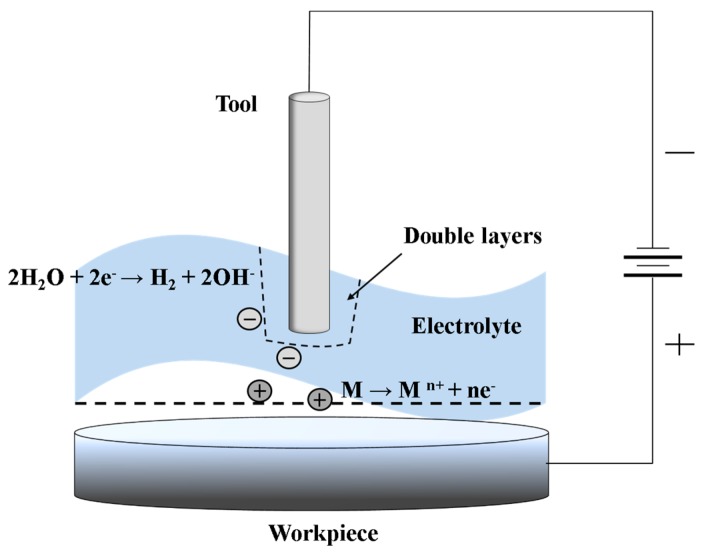
Principle of the electrochemical machining (ECM) process.

Figure 4 demonstrates the basic ECM process. ECM is the opposite of electrochemical coating or deposition processes. ECM can be thought of as a controlled anodic dissolution at the atomic level of a conductive workpiece by a shaped tool due to the flow of current between workpiece and tool. The anode material is electrochemically dissolved according to Faraday’s law and flushed away by the flowing electrolyte, together with the generated gas bubbles and Joule heat. In the ECM process, a tool is advanced into a workpiece, and the feed rate of the tool is the same as the rate of material removal [14]. 

In ECM, the required current is proportional to the desired rate of material removal, and a high-frequency short-pulse power supply can shorten the duration of the ECM machining current and decrease the thickness of the diffuse layer, which decreases the electron transfer rate. High-frequency power supply has already been investigated for enhancing anodic dissolution ability and decreasing the inter-electrode gap. The pulse duration of the machining current can be shortened to a few tens of nanoseconds, but the electrochemical process cannot achieve stationary conditions due to the extremely short pulse duration. During every pulse period, the electrochemical double layer at the interface of the electrode and electrolyte is gradually charged with increasing over-potential [14]. With the pulse current ends, the over-potential cannot reach the amplitude value according to short pulse duration. Therefore, the proper pulse duration depending on the different machining condition is a very important factor in ECM. With proper pulse duration and applied current, ECM can be used in Invar thin film hole machining, and precision geometry can be obtained. 

## 3. Results and Discussion

### 3.1. Wet Etching Experiments

**Figure 5:**
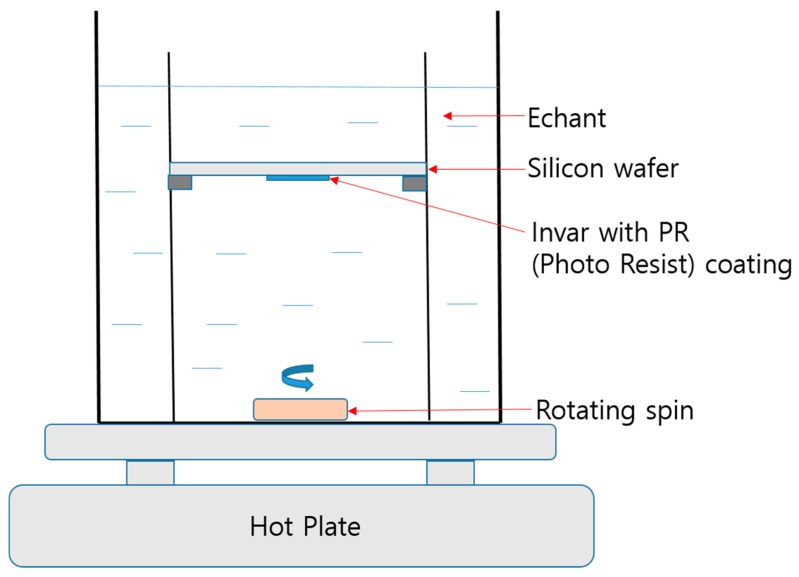
Schematic diagram of wet etching system.

An experiment was performed using a custom-built system designed specifically for dipping-type wet etching of Invar film. Figure 5 shows a schematic diagram of the system. Briefly, the Invar thin film (1 cm wide, 2 cm long) is attached to a 4-inch silicon wafer which is a fixing substrate and is fixed using a supporting jig in a reaction bath. The Invar film machining size can be increased by designing a large-scale system, and is not limited to the scale demonstrated here. 

The edge of the Invar thin film was sealed with polyimide tape on a flat silicon wafer to prevent the etchant from contacting the back face of the film. The etchant is 10 wt % FeCl_3_ solution with temperature fixed between 30 °C and 35 °C. To supply fresh etchant continuously, a stirrer (3-cm length) is rotated at the bottom of the reaction bath at 100 rpm. When the etchant concentration, temperature, and stirring speed increase, the material removal rate could be improved. However, with the increase of material removal rate, the adhesion between Invar film and DFR film sharply decreased. To obtain the desired hole machining geometry, it is not appropriate to increase the etchant concentration, temperature, and stirring speed. 

**Figure 6:**
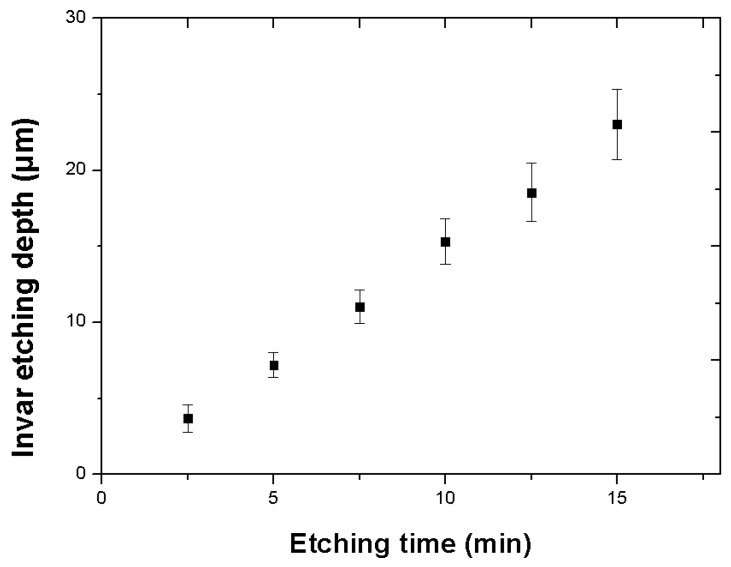
Invar film single side wet etching material removal rate.

**Figure 7:**
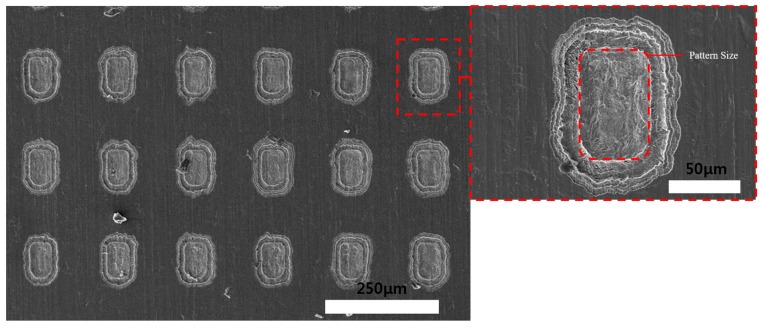
SEM images of Invar thin film after wet etching (photoresist coating face etching) for 5 min.

**Figure 8:**
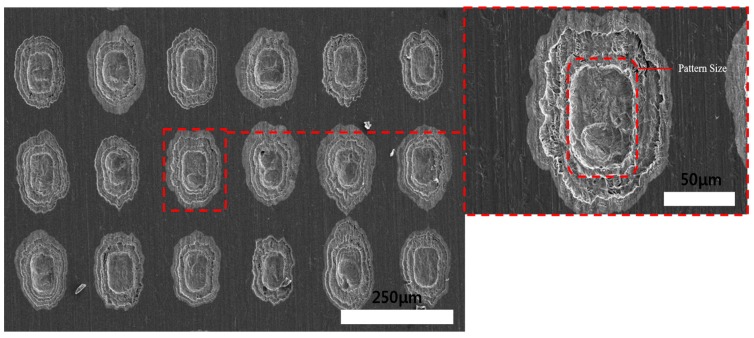
SEM images of Invar thin film after wet etching (photoresist coating face etching) for 10 min.

After the wet etching process, the DFR coating was removed using 10 wt % NaOH solution. Figure 6 shows the etching depth according to the etching time, which corresponds to the material removal rate etched on the DFR-coated face (about 1.5 µm/min). Theoretically, the array of holes could be machined after 20 to 25 min. Figure 7 and Figure 8 show SEM images of the Invar film after etching (on the coated face) for 5 and 10 min. Quadrilateral dimples were machined on the film after these times. The etching depth and machined area increased with the etching time, as shown in the figures. 

**Figure 9:**
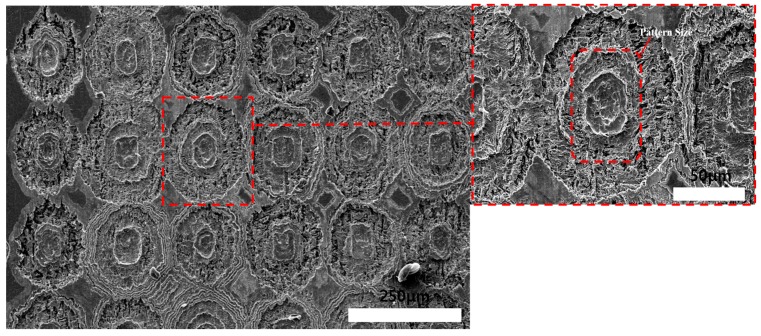
SEM images of Invar thin film after wet etching (photoresist coating face etching) for 15 min.

As mentioned, the isotropic etching led to undercutting. Therefore, the machined dimple size increased with the etching time. After 15 min of etching, the photoresist and Invar film separated before hole generation due to the undercutting, as shown in Figure 9. The edges of machined dimple areas overlapped due to the low adhesion between the DFR coating and Invar film. Generally, DFR coatings have poorer adhesion than liquid PR (Photo Resist) coatings. However, because of the small thickness of the Invar film, the flatness and coating productiveness are difficult to guarantee, which makes DFR coating more appropriate. The machined surface quality of the dipping-etched Invar film is not good enough for OLED shadow masks, which require high quality to avoid excessive organic compounds and decreased lifetime of the shadow mask, which is significant in manufacturing. The surface quality of machined Invar could be improved by using spray-type wet etching instead of dipping-type, which can improve the efficient and uniform etchant supply, but the adhesion problem between Invar and DFR is difficult to solve in small pitch shadow mask, because of isotropic wet etching machining characteristics.

### 3.2. Wet Etching and Electrochemical Fusion Machining

**Figure 10:**
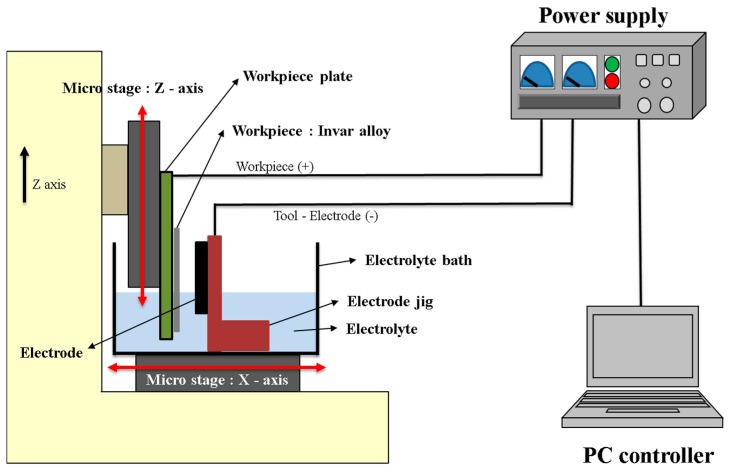
Schematic diagram of the electrochemical machining system.

The Invar thin film photoresist ECM system includes a micro-pulse power supply with a voltage range of 0.5 to 30 V, maximum current of 100 A, pulse on-time of 1 μs to 990 ms, and pulse off-time of 10 μs to 990 ms. The applied current, voltage, machining time, and pulse duration were controlled by a PC. The electrolyte bath was made of Teflon to protect it from corrosion by the alkaline and acidic electrolytes. The position of the workpiece and electrode was controlled by micro-stages. The minimum movement of the micro-stage was 10 μm per step. A schematic diagram of the ECM system is shown in Figure 10. A rectangular Invar thin film with photoresist coating was fixed on the workpiece plate on a Teflon base and immersed into the electrolyte bath. The electrode was a flat plate made of stainless steel 304, which was fixed in a Teflon electrode jig and used as a cathode for machining the Invar thin film. The position of the cathode could be adjusted by the micro-stage along the X-axis to control the inter-electrode gap during ECM. 

Table 1 shows the ECM conditions for photoresist-coated Invar thin film. The workpiece was coated with negative-type photoresist and 30 μm thickness. The inter-electrode gap was fixed as 1 mm, and the electrolyte was a solution containing 3 M of NaCl and 1 M of glycerin solution. The applied voltage was 5 V, the pulse on-time was 9 µs, and off-time was 27 µs. The inter-electrode gap, electrolyte, and pulse on/off time were determined by previous research. In ECM, the inter-electrode gap influences the current density, which is important in the material removal rate. A small inter-electrode gap can obtain relatively high current density, which can increase the material removal rate. However, a small inter-electrode gap can influence the effective removal of ions and bubbles generated from electrochemical reaction, and we found that a 1-mm inter-electrode gap was appropriate for photoresist ECM. The productivity of ECM could be faster than wet etching if the applied voltage and pulse on/off time were appropriate and high enough. However, because of current distribution characteristics, high voltage and high duration of pulse on-time photoresist ECM results in the separation of Invar and DFR film, and also results in elliptical dimple or hole shape instead of quadrilateral shape. Therefore, 5 V and pulse on-time 9 µs/off-time 27 µs are appropriate for micro hole array machining of Invar thin film. 

**Figure 11:**
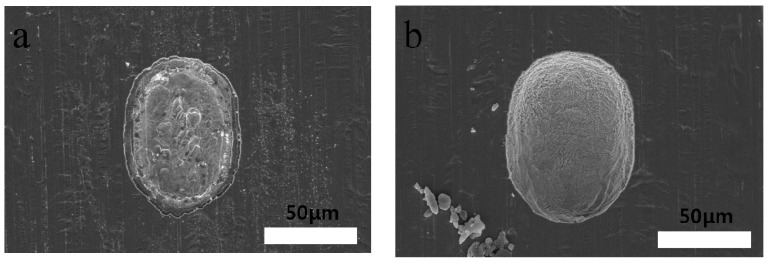
SEM images of Invar film machined surface produced by (**a**) wet etching, (**b**) electrochemical machining.

Figure 11 shows SEM images of the machined Invar film surface with wet etching and ECM. Figure 11a shows the surface quality of the dipping-type wet-etched Invar, and Figure 11b shows the surface quality after ECM. The machining time was 1 min in both cases. The figure shows that the Invar surface quality is better after ECM than that after wet etching. With wet etching, the surface of the hole edge is not smooth enough because of the uncontrollable undercutting characteristics. Additionally, the dissolved metal ions are difficult to remove quickly in etching, which could lead to poor machined surface quality. This machining characteristics of wet etching are not appropriate for obtaining an accurate and smooth surface on Invar. In contrast, the dissolved metal ions are removed by fresh electrolyte and the bubbles generated during ECM, and fresh electrolyte is supplied for the electrochemical reaction, which is why the surface quality is better after ECM. 

**Figure 12:**
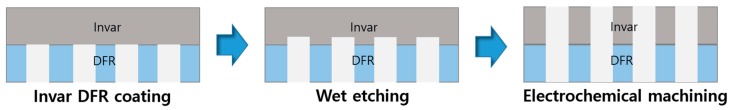
Wet etching and electrochemical fusion machining (photoresist coating face etching).

**Figure 13:**
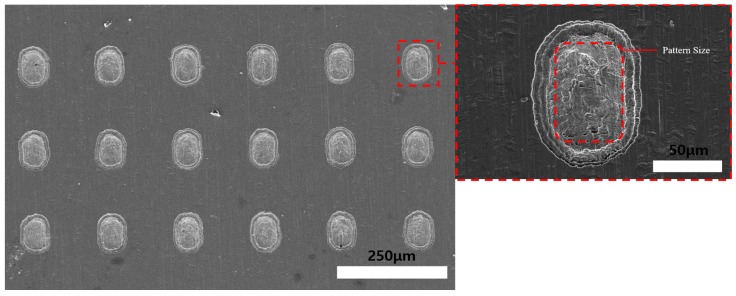
SEM images of Invar thin film after wet etching for 2 min (photoresist coating face etching).

**Figure 14:**
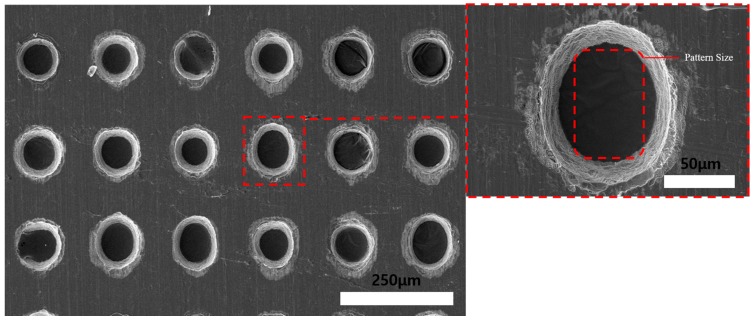
SEM images of 30 min electrochemical machined Invar thin film after wet etching (photoresist coating face etching) for 2 min.

**Figure 15:**
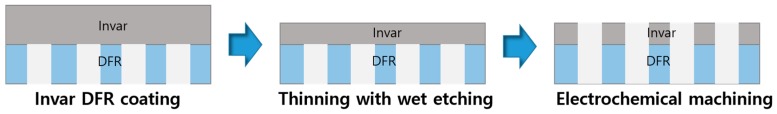
Wet etching and electrochemical fusion machining (photoresist coating opposite face etching).

In ECM, the material removal rate can be very high if the applied current is high enough. However, in photoresist precision ECM, because of the current distribution, high applied current and long machining time results in elliptical holes instead of quadrilateral geometry. This is the reason why material removal in photoresist ECM is at a very low level compared to photoresist wet etching method. Therefore, the wet etching and electrochemical fusion machining methods were carried out to improve the machining geometry, surface quality, and decrease the machining time. The fusion machining was done with wet etching on the photoresist-coated face and the uncoated face before ECM. Figure 12 shows the process with the Invar film coated-face etching. The wet etching was carried out for 2 min, as shown in Figure 13. To reduce the undercutting phenomenon in wet etching, the wet etching time was controlled as 2 min. After that, ECM was carried out with the conditions in Table 1, and the machining time was 30 min. Figure 14 shows that the edge surface quality of the holes was improved after ECM. However, the hole geometry uniformity is difficult to control when the electrochemical machining is maintained for a long period with this method. Additionally, the machined hole image was elliptical instead of quadrilateral because of the electrochemical machining current distribution characteristics. Therefore, to decrease the electrochemical machining time, wet etching on the photoresist’s uncoated face was carried out to thin the Invar thin film. The process is shown in Figure 15. The thickness of the Invar film decreased as the etching time increased. 

**Figure 16:**
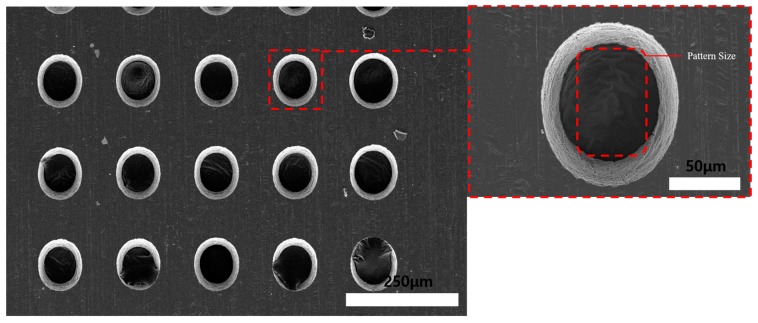
SEM images of 30 min electrochemical machined Invar thin film after wet etching (photoresist coating opposite face etching) for 2 min.

Figure 16 shows SEM images of the 30 min ECM Invar film after 2 min of wet etching on the uncoated face. With increased etching time, the thickness of the Invar film decreased. A thicker film requires more time to machine holes with ECM, which results in elliptical holes because of the ECM current distribution characteristics. The Invar film should be thin enough to obtain hole shapes that are similar to the shapes in the photoresist coating. 

**Figure 17:**
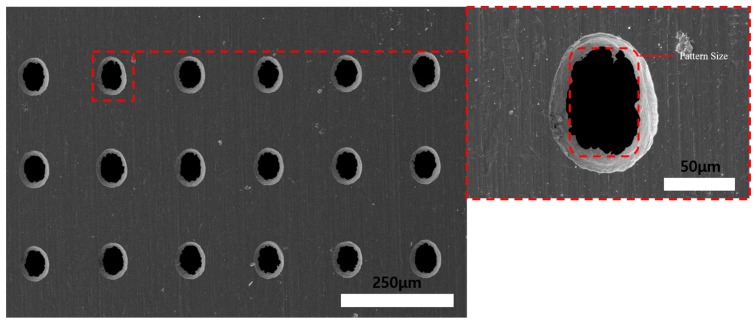
SEM images of 15 min electrochemical machined Invar thin film after wet etching (photoresist coating opposite face etching) for 5 min.

**Figure 18:**
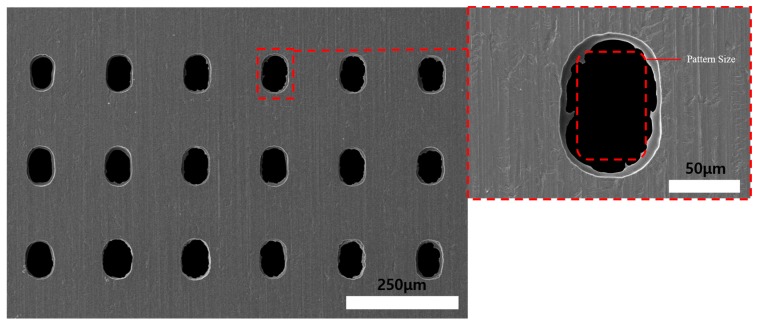
SEM images of 5 min electrochemical machined Invar thin film after wet etching (photoresist coating opposite face etching) for 10 min.

Figure 17 shows SEM images of the 15 min ECM Invar film after 5 min of wet etching. The machined hole quadrilateral geometry increased as the Invar thickness decreased. Additionally, by the fusion machining method, the total machining time could be decreased. After 10 min of wet etching on the non-photoresist-coated face and 5 min ECM on the photoresist-coated face, high accuracy of the quadrilateral hole shape was achieved, as shown in Figure 18. The experimental results show that the thin Invar film increased the machining accuracy of the holes. However, the uniformity of the Invar film thinning is difficult to control in dipping-type wet etching. With the increase of wet etching thinning time, the Invar film thickness tolerance increased, which could distort the electrical distribution during ECM and reduce the machining geometry accuracy. An etching time of 10 min was found to be appropriate for uniform thinning and obtaining quadrilateral geometry holes. The Invar film could be thinner if the wet etching time was increased. However, if the Invar film thickness is too small, the control of the thin film mask during frame process could be very difficult. Therefore, a 15 μm thickness Invar film after the wet etching thinning process is appropriate. The Invar surface quality after ECM was better than that after coated-face wet etching. Additionally, the machining geometry could be improved by the fusion machining. Using ECM after wet etching on DFR uncoated face with 60 × 90 μm DFR patterned 30 μm thickness Invar film experiment results obtained a 75 ± 5 × 105 ± 5 μm uniform quadrilateral hole array. With different DFR patterned size, different hole arrays could be obtained.

The wet etching and photoresist ECM fusion machining is a promising machining method for Invar thin film hole machining, as it can improve the hole geometry and surface quality compared to the wet etching method. 

## 4. Conclusions

This study investigated single-side wet etching and a fusion method of wet etching and ECM for machining holes on photoresist-coated Invar thin film. Because of isotropic machining characteristics in wet etching, the dry film photoresist and Invar film separated with increased etching time, making it difficult to control the machined hole geometry. This also occurred in dipping-type wet etching; because of the bad flow field, the machined surface quality is not good enough. 

However, in ECM the dissolved metal ions and bubbles can be removed by electrolyte flow, and fresh electrolyte is supplied. This results in better surface quality of the ECM Invar film than in the wet-etched film. This means that we could use ECM to improve the surface quality after wet etching. Additionally, the undercutting characteristics can be reduced by optimizing the applied voltage and pulse on/off time in ECM. 

When wet etching on the photoresist-coated face, the edges of holes could be improved after ECM. However, the ECM hole uniformity is difficult to control with this method. Because of the long ECM time and current distribution characteristics, the holes attain an elliptical geometry. Therefore, this fusion method is not appropriate for obtaining quadrilateral hole geometry.

When etching on uncoated face to thin the Invar film before ECM, the machined quadrilateral hole geometry increased as the film thickness decreased. However, the uniformity of the decreasing thickness is difficult to control in wet etching. The Invar film thickness tolerance increased with the increase of wet etching time. An etching time of 10 min on the uncoated face was found to be appropriate for thinning uniformity and hole machining geometry. By using this method, highly accurate quadrilateral hole geometry could be obtained. The fusion method opens up the possibility of improving the efficiency and machining hole geometry for OLED shadow mask fabrication.

## Figures and Tables

**Table 1:** ECM conditions for photoresist coating Invar thin film.

Conditions	Values
Workpiece	Invar thin film 30 μm
Electrode	Stainless steel 304 plate
Voltage	5 V
Inter-electrode gap	1 mm
Pulse on-time	9 μs
Pulse off-time	27 μs
Machining time	5–30 min
Electrolyte	3 M NaCl 1 M Glycerine
